# A Ligand Peptide Motif Selected from a Cancer Patient Is a Receptor-Interacting Site within Human Interleukin-11

**DOI:** 10.1371/journal.pone.0003452

**Published:** 2008-10-20

**Authors:** Marina Cardó-Vila, Amado J. Zurita, Ricardo J. Giordano, Jessica Sun, Roberto Rangel, Liliana Guzman-Rojas, Cristiane D. Anobom, Ana P. Valente, Fábio C. L. Almeida, Johanna Lahdenranta, Mikhail G. Kolonin, Wadih Arap, Renata Pasqualini

**Affiliations:** 1 The University of Texas M. D. Anderson Cancer Center, Houston, Texas, United States of America; 2 National NMR Center, Federal University, Rio de Janeiro, Brazil; University of Helsinki, Finland

## Abstract

Interleukin-11 (IL-11) is a pleiotropic cytokine approved by the FDA against chemotherapy-induced thrombocytopenia. From a combinatorial selection in a cancer patient, we isolated an IL-11-like peptide mapping to domain I of the IL-11 (sequence CGRRAGGSC). Although this motif has ligand attributes, it is not within the previously characterized interacting sites. Here we design and validate in-tandem binding assays, site-directed mutagenesis and NMR spectroscopy to show (i) the peptide mimics a receptor-binding site within IL-11, (ii) the binding of CGRRAGGSC to the IL-11Rα is functionally relevant, (iii) Arg^4^ and Ser^8^ are the key residues mediating the interaction, and (iv) the IL-11-like motif induces cell proliferation through STAT3 activation. These structural and functional results uncover an as yet unrecognized receptor-binding site in human IL-11. Given that IL-11Rα has been proposed as a target in human cancer, our results provide clues for the rational design of targeted drugs.

## Introduction

Phage display enables identification of tissue-specific or angiogenesis-related molecular signatures on blood vessels and thereby allows ligand-directed delivery [1]–[10]. Previously we showed, through direct screening of a combinatorial peptide library in a cancer patient, that the selective homing peptides localized non-randomly to specific organs [1]; we also identified a cyclic peptide (sequence CGRRAGGSC) targeting prostate vasculature and prostate cancer as an interleukin-11 (IL-11) mimic [1], [2]. In a different line of investigation, Kang et al. [11] proposed a central role for the IL-11 pathway in the genetic progression of malignant tumors metastatic to bone. Activation of members of the signal transduction and activator of transcription (STAT) family, in particular STAT3, was revealed downstream as a consequence of the binding of IL-11 to its corresponding receptor [12].

Although IL-11 was initially characterized as a cytokine with thrombopoietic activity, it was later found to have pleiotropic effects in multiple tissues [13]. Recombinant IL-11 is an FDA-approved drug (oprelvekin; Neumega®) used against chemotherapy-induced thrombocytopenia (http://www.fda.gov/cder/biologics/products/opregen112597.htm) [14]. Along with more than 10 other four-helix bundle cytokines, including interleukin-6 (IL-6), leukemia inhibitory factor (LIF), ciliary neurotrophic factor (CNTF), oncostatin-M (OSM), and cardiotrophin-1 (CT-1), IL-11 belongs to the gp130 or IL-6-type [15], [16]. These cytokines elicit responses by the assembly of oligomeric signaling complexes that contain the transmembrane receptor gp130. The structure of IL-11 has been the subject of molecular modeling and mutagenesis studies [17]–[21]. Research has revealed that IL-11 contains three known receptor-binding sites (I, II and III). Site I of IL-11 binds to the cytokine-receptor homology region (CHR) in domains 2 and 3 (D2-D3) of IL-11Rα, whereas sites II and III interact with two separate areas of the gp130 homodimer: the CHR (D2-D3, site II) and the Ig-like domain (D1, site III) [22]–[24]. However, no direct structural analysis is available for either IL-11 or the signaling complex. In fact, while the X-ray structure of the IL-6 receptor complex as a hexameric IL-6/IL-6Rα/gp130 ternary complex has been introduced as a model for IL-11 [25]–[27], the accuracy of this extrapolation has actually been challenged because IL-11 and IL-6 share no significant similarity in primary structure and interact with different receptors presumably through different mechanisms [28]. A ligand-induced transition from an active tetrameric complex (including a gp130 dimer) to an inactive hexameric complex has been proposed for IL-11 [28], [29]. Responsiveness to IL-11 is restricted to cells expressing IL-11Rα in addition to gp130. In analogy to the IL-6 receptor α (IL-6Rα) and IL-6, it has been postulated that IL-11Rα “presents” IL-11 to gp130, which is recruited as a homodimer and leads to the generation of a so-called high affinity IL-11 receptor complex. This active complex is the initiating step in the activation of Janus kinase (JAK)/Tyk tyrosine kinases, which in turn phosphorylate tyrosine residues in the cytoplasmic region of gp130; these subsequently serve as docking sites for members of the STAT family of transcription factors, e.g., STAT3 [12], [13], [17].

Although the in vivo-selected homing motif CGRRAGGSC had certain ligand-specific attributes [1], [2], the corresponding location within the native cytokine is not in an established interacting site between IL-11 and IL-11Rα [17]–[28]. We hypothesized and subsequently confirmed that this peptide motif functions as a new binding site within IL-11. Tandem site-directed mutagenesis, binding assays, nuclear magnetic resonance (NMR) spectroscopy, and signal transduction analysis strongly support the conclusion that this peptide sequence is indeed a previously unrecognized receptor-interacting site within human IL-11.

## Results

### Selected sequence-binding to the IL-11 receptor complex is specific and does not include gp130

**Figure 1:**
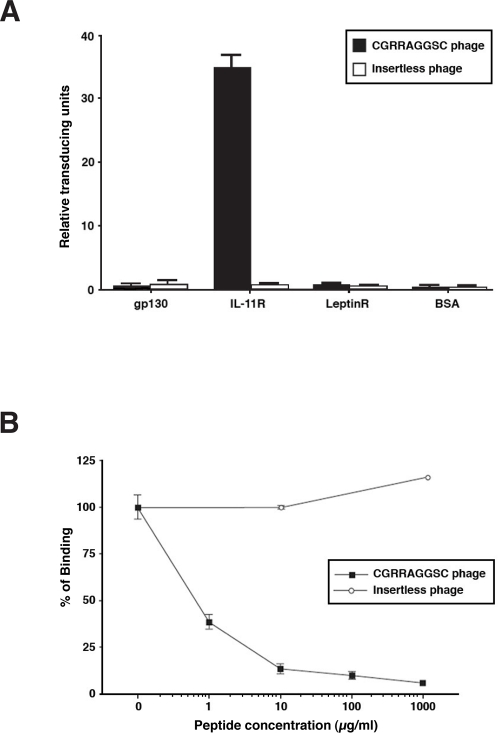
Binding of IL-11-like peptides to IL-11Rα. (A) CGRRAGGSC-phage binding to the individual receptor components of the IL-11 receptor complex: IL-11Rα and gp130. Leptin receptor and BSA served as negative controls for binding. (B) Concentration-dependent binding inhibition of CGRRAGGSC-phage to IL-11Rα by the cognate synthetic peptide. Bars represent mean±standard error of the mean (SEM).

We have previously established that phage displaying the motif CGRRAGGSC specifically interacts with IL-11Rα; this interaction was inhibited by recombinant IL-11 in a concentration-dependent manner but not by IL-1β [1], [2]. Because the functional IL-11 receptor complex appears to be a multi-heterodimer composed of IL-11, IL-11Rα, and gp130 [15], we first evaluated whether the selected IL-11-like motif [1], [2] would bind to IL-11Rα, to gp130, or to both. For this purpose, we coated plates with the following protein: IL-11Rα, gp130, or the leptin receptor (an unrelated gp130 partner). Bovine serum albumin (BSA) served as a negative control for the binding assay (Figure 1A). CGRRAGGSC-phage did not bind above background levels to immobilized gp130, in comparison to its significant binding to human IL-11Rα (Figure 1A), data indicating no phage binding to gp130 alone. Moreover, CGRRAGGSC-phage binding to the leptin receptor or to BSA was undistinguishable from that of untargeted control phage. These results establish that the CGRRAGGSC peptide only binds to IL-11Rα in the receptor complex when individual subunits are analyzed. We next showed that CGRRAGGSC-phage binding to IL-11Rα is mediated by the IL-11-like motif, because synthetic CGRRAGGSC inhibited binding of the cognate phage in a concentration-dependent manner (Figure 1B).

### NMR spectroscopy of the targeted peptide-receptor interaction

NMR is a particularly suitable methodology to study medium-to-low affinity binding [30], [31], which is often the case for the interaction between peptide ligands identified by phage display (such as CGRRAGGSC peptide and IL-11Rα). By measuring subtle changes in the NMR parameters of the ligand (such as chemical shifts and relaxation times) one can probe binding events that occur at the µM to mM concentration range due to the fast exchange between the bound conformation and free form of the ligand. Once the fast exchange regiment is reached an excess of ligand (mM) over the receptor (µM) can be employed for the measurements. Fast exchange condition is frequently present in binding studies involving peptides selected from phage display libraries because of their inherent medium to low affinity range [30]–[32]. Thus, to characterize the structural basis of the interaction between the peptide CGRRAGGSC and IL-11Rα, we applied NMR-spectroscopy [30], [32]. We began by analyzing the structural behavior of the free synthetic peptide CGRRAGGSC by proton NMR. The one-dimensional (1D-^1^H-NMR) spectrum displayed broad lines at 25°C (Figure S1A) indicating the presence of conformational exchange among multiple CGRRAGGSC conformers. Although the resonance line became more defined at 5°C, conformational exchange persisted at low temperatures (Figure S1A); indeed, the occurrence of multiple conformers is not uncommon in this setting and has not precluded the study of ligand receptor interactions [30]. In the case of CGRRAGGSC peptide almost all the resonances in the spectra were unambiguously assigned to the individual residues of the peptide based on two-dimensional proton spectra (2D-^1^H-NMR) TOCSY and NOESY (Table S1 and S2).

**Figure 2:**
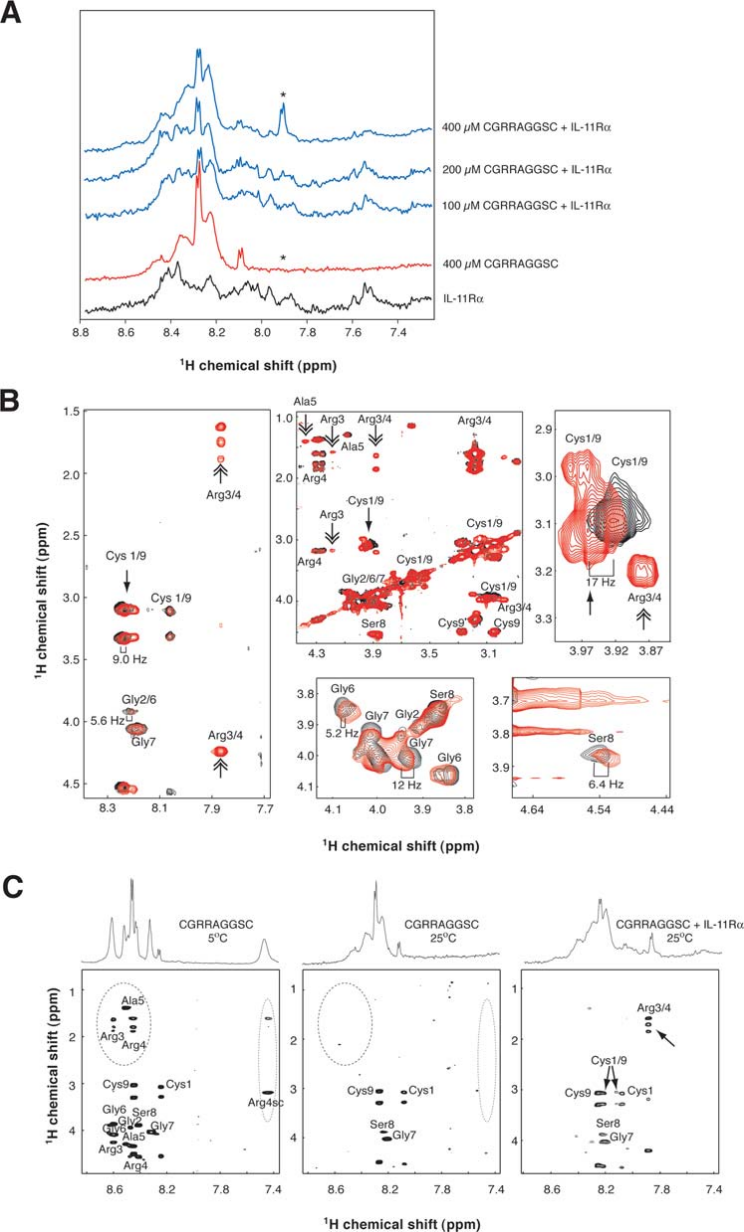
Structural basis of the interaction between CGRRAGGSC and IL-11Rα. (A) Effect of the CGRRAGGSC peptide concentration upon biding to IL-11Rα. Amide region of 1D-^1^H-NMR spectra of increasing molar concentrations of the peptide CGRRAGGSC binding to IL-11Rα (6 µM) is shown (blue). CGRRAGGSC peptide alone (400 µM) and the spectrum of IL-11Rα alone are also shown (in red and black, respectively). The appearance of arginine side-chain resonances (not seen in the spectrum of the peptide alone) is indicated (*). (B) Chemical shift changes induced on the CGRRAGGSC resonances by binding to IL-11Rα. The 2D-^1^H-NMR TOCSY spectra of CGRRAGGSC (400 µM) either alone (black) or in the presence of 6 µM IL-11Rα (red) are shown. Single-headed and double-headed arrows indicate chemical shift changes and the appearance of new spin-systems, respectively. (C) Composition of amide region of 1D-^1^H-NMR and corresponding 2D-^1^H-NMR TOCSY spectra of the CGRRAGGSC peptide at 5°C (left), at 25°C (middle) and at 25°C in the presence of IL-11Rα (right). The circles with dotted lines and the arrow indicate arginine resonances.

Having characterized the structural behavior of the CGRRAGGSC peptide in solution and assigned its resonances in the NMR spectra, we next set up binding assays [30] to gain insight into the basis for receptor binding to the ligand peptide. The spectrum of free CGRRAGGSC was compared to those of CGRRAGGSC in the presence of IL-11Rα (under a molar excess of peptide of ∼16-, 33- and 66-fold), and changes in chemical shift (Δδ) were analyzed (Figure 2A). Although the precise mapping of the residues in CGRRAGGSC could not be achieved from the 1D-^1^H-NMR spectra due to receptor peak overlap, the interaction between CGRRAGGSC and IL-11Rα resulted in chemical shift alterations and new spin systems (Figures 2A–C). Such changes are indicative of binding and are suggestive that multiple residues contribute to the interaction. Moreover, the binding equilibrium was dependent on peptide concentration and consistent with a fast exchange rate (µs range) due to the resonances of free and bound forms of the peptide were detected as average bands (Figure 2A).

To calculate the individual chemical shift changes (Δδ) induced by IL-11Rα upon each of the hydrogen atoms of CGRRAGGSC, we compared the individual 2D-^1^H-NMR TOCSY spectra obtained in the absence and presence of IL-11Rα (molar peptide excess ∼66-fold) (Figure 2B and Table 1). These spectra were also used for the analysis of new spin systems (Figures 2B and 2C). In the 2D-^1^H-NMR TOCSY the receptor NMR signal was filtered during the 80 ms spin lock, resulting in a cleaner analysis of chemical shift perturbation. We observed that most individual resonances in CGRRAGGSC showed significant Δδ upon binding to IL-11Rα (Figure 2B and Table 1), a result indicating that nearly all residues within CGRRAGGSC participate in the binding to the receptor in a direct or indirect manner. The most prominent Δδ (>10 Hz) involved the α-hydrogen atoms of the Gly^7^ and of the Cys residues (which could not be unambiguously assigned and here is referred to as Cys^1/9^). However, Δδ>5 Hz for residues Cys^1^, Gly^2^, Gly^6^, Ser^8^, and Cys^9^ were also noteworthy. The new spin systems observed in the 1D-^1^H-NMR spectra (Figure 2A) also appeared in the TOCSY spectra, and were assigned to Cys^1/9^, Arg^3/4^, and Ala^5^ (Figure 2B, double-headed arrows). These new spin systems map to the peptide region that undergoes a complex conformational equilibrium in the free state. This result suggests that, upon binding to IL-11Rα, the segment Cys-s-s-Cys-Gly-Arg-Arg-Ala in CGRRAGGSC becomes conformationally constrained, resulting in a gain-of-structure and corresponding loss-of-freedom for CGRRAGGSC in the bound state. Consistently, similar results were observed by comparing the 1D-^1^H-NMR and the 2D-^1^H-NMR TOCSY spectra of CGRRAGGSC at 5°C and 25°C (Figure 2C). Arg^3^, Arg^4^ and Ala^5^ resonances could only be detected in the spectra at 5°C (Figure 2C, left panel) but not in the spectra at 25°C (Figure 2C, middle panel), again indicative that conformational variability plays a role when detecting such spin systems which has been recently reported [31], [33]–[35]. However, upon binding to IL-11Rα, resonances corresponding to Arg^3^, Arg^4^ and Ala^5^ were also observed in the spectra at 25°C (Figure 2C, right panel).

**Table 1:** Individual chemical shift changes in CGRRAGGSC upon interaction with IL-11Rα.

Residue	Hydrogen	Δδ (Hz)
**Cys^1^**	HN	2.1
	Hα	2.1
	Hβ2	0.0
	Hβ3	***−8.9***
**Cys^1/9^**	HN	N.D.
	Hα	***−17.0***
	Hβ	N.D.
**Gly^2^**	HN	N.D.
	Hα2	***5.8***
	Hα3	N.D.
**Arg^3^**	HN	N.D.
	Hα	1.0
	Hβ2	−2.8
	Hβ3	−3.1
	Hγ	−1.0
	Hδ	−4.1
**Arg^4^**	HN	N.D.
	Hα	−2.7
	Hβ2	−2.8
	Hβ3	−3.1
	Hγ	−1.0
	Hδ	−4.1
**Ala^5^**	HN	N.D.
	Hα	−2.4
	Hβ	0
**Gly^6^**	HN	***5.8***
	Hα2	***5.2***
	Hα3	***5.0***
**Gly^7^**	HN	3.0
	Hα2	***>12***
	Hα3	0
**Ser^8^**	HN	N.D.
	Hα	***6.4***
	Hβ	−1.2
**Cys^9^**	HN	***9.0***
	Hα	−1.6
	Hβ1	−1.8
	Hβ2	−1.0

Differences in chemical shift change (Δδ = δ_free_−δ_bound_) were determined in free CGRRAGGSC (400 µM) and in the presence of IL-11Rα (6 µM) calculated from 2D-^1^H-TOCSY. Bold and italicized numbers represent chemical shift changes greater than 5 Hz. Labile resonances were not detected (N.D.) due to fast solvent exchange or receptor peak overlap.

Taken together, our NMR data indicate that conformational variability plays a structural role in CGRRAGGSC peptide interaction with IL-11Rα and suggest that: (i) most residues in CGRRAGGSC change their conformation upon binding to IL-11Rα, (ii) the Cys-s-s-Cys-Gly-Arg-Arg-Ala domain of the peptide undergoes a receptor-dependent gain-of-structure, (iii) the motif Gly-Gly-Ser-Cys-ss-Cys encompasses a candidate site for direct interaction of the peptide with IL-11Rα, and (iv) that the residues Gly^7^ and Ser^8^, in agreement with the mutational studies (described below), are also relevant for binding.

### Functional analysis of wild-type and site-directed IL-11 mutants

In previous work, other investigators have produced recombinant proteins containing point mutations to unveil functional attributes of the native IL-11; notably, point mutations within the site containing the CGRRAGGSC sequence have not been reported [17]–[21]. Thus, to further demonstrate that residues 112–117 represent a protein-interacting site, we first attempted to generate a series of recombinant site-directed alanine-scan mutants of the motif RRAGGS within human IL-11.

**Figure 3:**
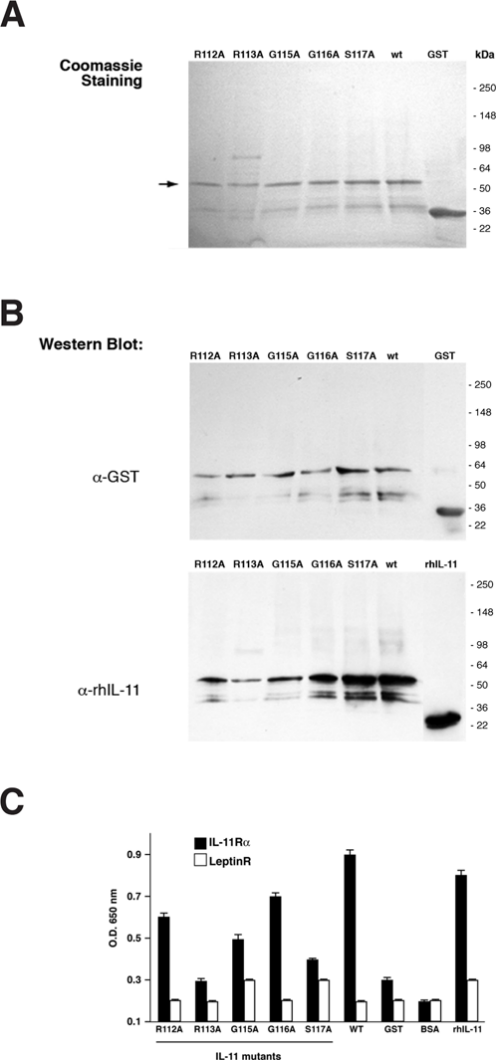
Binding of wild-type or site-directed mutants of IL-11 to IL-11Rα. (A) Purified recombinant proteins were analyzed by Coomassie staining. (B) Western blot analyses with polyclonal anti-IL-11 and anti-GST antibodies. (C) Recombinant GST fusion proteins (alanine scan mutants of residues 112–117 of IL-11, wild-type IL-11, GST alone, or rhIL-11) were coated in triplicate overnight and incubated with IL-11Rα. Binding was detected with anti-Fc antibody. Bars represent mean±standard error of the mean (SEM).

Whereas DNA sequencing (data not shown) and SDS-PAGE analysis (Figure 3A) showed that the wild-type and mutant IL-11 recombinant proteins were produced with the expected molecular weight, a number of IL-11 mutants presented steric hindrance and misfolding aberrances. Such inherent technical limitation of any mutational study is evident from the lack of epitope accessibility observed in studies with anti-IL11 antibodies (Figure 3B and data not shown). Despite these potential issues, we next used ELISA to assess the attributes of IL-11 point mutants (residues 112–117) for their ability to bind to the IL-11Rα. Mutation of the residues Arg^113^ (R113A), Gly^115^ (G115A), and Ser^117^ (S117A) markedly reduced binding of IL-11 to the receptor, relative to the wild-type sequence (86, 71, and 86% binding inhibition, respectively), whereas mutation of residues Arg^112^ (R112A) and Gly^116^ (G116A) produced only a moderate effect (43 and 29% binding inhibition, respectively) (Figure 3C). These data indicate that most of the residues within the RRAGGS motif participate in the binding to IL-11Rα; however, Gly^115^ and Ser^117^ are likely the key residues mediating the ligand-receptor interaction. Thus, consistent with our working hypothesis, the alanine-scan mutants did show reduced, albeit specific, binding to the corresponding receptor, IL-11Rα (Figure 3C).

The role of residue Arg^113^ is somewhat problematic to evaluate from ELISA, because this mutation affected the reactivity of the anti-IL-11 antibodies. To rule out the possibility that the observed reduction in binding of the IL-11 mutants to the IL-11Rα was secondary to misfolding of the full-length protein and/or steric hindrance, we devised an alternative ligand-receptor assay based on site-directed mutagenesis of peptide-targeted phage. Single-residue alanine scanning mutagenesis of the CGRRAGGSC phage were produced and binding of each phage to immobilized IL-11Rα was tested in a functional assay (Table 2). Mutation of Arg^4^, Gly^7^ and Ser^8^ residues in CGRRAGGSC phage abrogated binding to the receptor (Table 2), whereas mutation of residues Arg^3^ and Gly^6^ did not inhibit binding but reduced the CGRRAGGSC-receptor interaction by over 70%. These data are in agreement with the NMR and the IL-11 site-directed mutagenesis studies and indicate that most residues within the RRAGGS motif participate in the interaction with IL-11Rα. Moreover, the data identify a central role for the Ser^8^ residue in CGRRAGGSC (corresponding to the Ser^117^ of IL-11) in binding to the receptor and indicate that both glycine residues are important for interaction with IL-11Rα. In conclusion, residues 112–117 in human IL-11 comprise a structural and functional site for the interaction of the protein with its receptor.

**Table 2:** Phage binding to IL-11Rα.

Peptide Sequences	Relative Binding^a^
CGRRAGGSC^b^	**+++**
C**A**RRAGGSC	**++**
CG**A**RAGGSC	**+**
CGR**A**AGGSC	**−**
CGRRA**A**GSC	**+**
CGRRAG**A**SC	**−**
CRGSGAGRC^c^	**−**
Negative control	**−**

a Ratio of Binding to IL-11Rα/Binding to BSA: **+++**: >30; **++**: 15–30; **+**: 6–15; **−**: <6.

b Positive control.

c Scrambled control; Negative control, insertless.

### The synthetic peptide CGRRAGGSC induces cell proliferation

**Figure 4:**
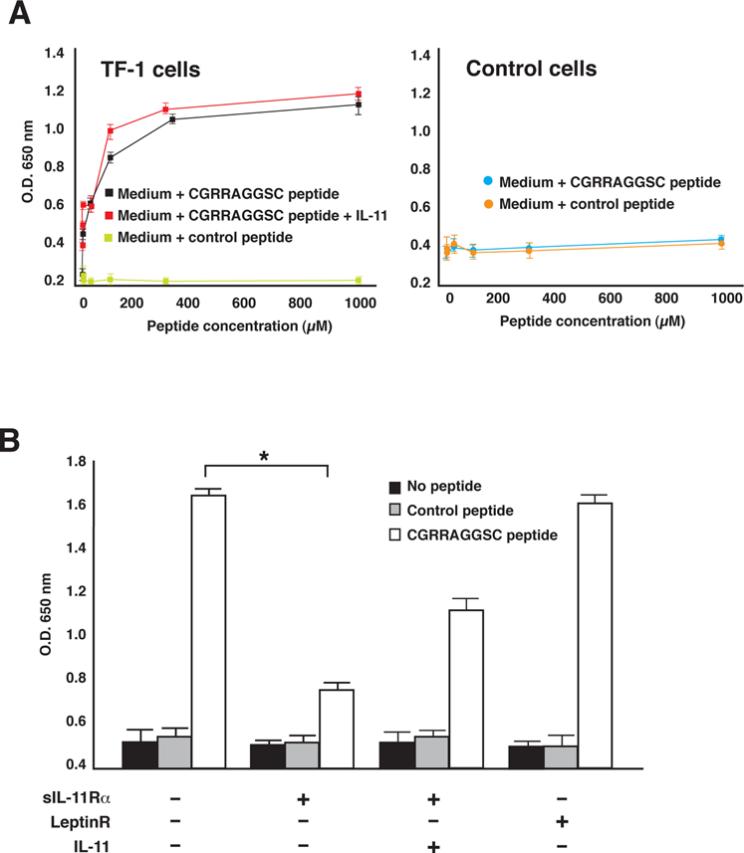
Biological effects of the IL-11-like peptide CGRRAGGSC on IL-11-responsive cells. (A) Concentration-dependent proliferative response to CGRRAGGSC is shown on IL-11Rα-expressing human TF-1 leukemia cells in the absence or presence of IL-11 (left panel). No response is observed on non-IL-11Rα-expressing control cells (right panel). (B) Soluble IL-11Rα-mediated inhibition of the proliferative effect induced by 150 µM CGRRAGGSC peptide (and by IL-11). * t-test, P<0.005. Bars represent mean±standard error of the mean (SEM).

Having shown that the motif RRAGGS represents a relevant site within human IL-11, we proceeded to determine whether the corresponding synthetic peptide is biologically active. IL-11 induces concentration-dependent proliferation when incubated with human TF-1 leukemia cells ([36]; and data not shown). Thus, we evaluated whether the corresponding synthetic CGRRAGGSC peptide mimics IL-11 in its stimulation of these cells upon binding to the IL-11Rα. We first exposed TF-1 cells to the CGRRAGGSC peptide in the presence or absence of either IL-11 or GM-CSF (positive control) under pre-determined conditions in which IL-11 induces optimal cell proliferation. Soluble CGRRAGGSC peptide induced a potent growth-stimulatory effect on TF-1 cells, alone and in the presence of IL-11 (Figure 4A, left panel) or GM-CSF (data not shown), whereas no effect was observed on control (non-IL-11Rα-expressing) cells (Figure 4A, right panel) [2]. This peptide-induced proliferative effect was more pronounced for CGRRAGGSC plus IL-11 or CGRRAGGSC alone than for CGRRAGGSC plus GM-CSF (data not shown); an unrelated cyclic control peptide did not show detectable effects by itself or in combination with IL-11. Next, to evaluate whether CGRRAGGSC-induced proliferation is dependent on cell surface IL-11Rα, we used the corresponding soluble receptor, sIL-11Rα, as a molecular decoy for peptide binding. Cell proliferation induced by CGRRAGGSC was lower in the presence of sIL-11Rα (t-test, P<0.005) but not in the presence of a control soluble receptor (Figure 4B). Of note, less inhibition was observed when cells were cultured with CGRRAGGSC, sIL-11Rα and IL-11, data indicating that CGRRAGGSC might interact with the complex IL-11-sIL-11Rα (Figure 4B). These results show that the synthetic peptide CGRRAGGSC can induce cell proliferation by itself, possibly acting as an IL-11Rα-stimulatory ligand.

### STAT3 phosphorylation mediates CGRRAGGSC-induced cell proliferation

**Figure 5:**
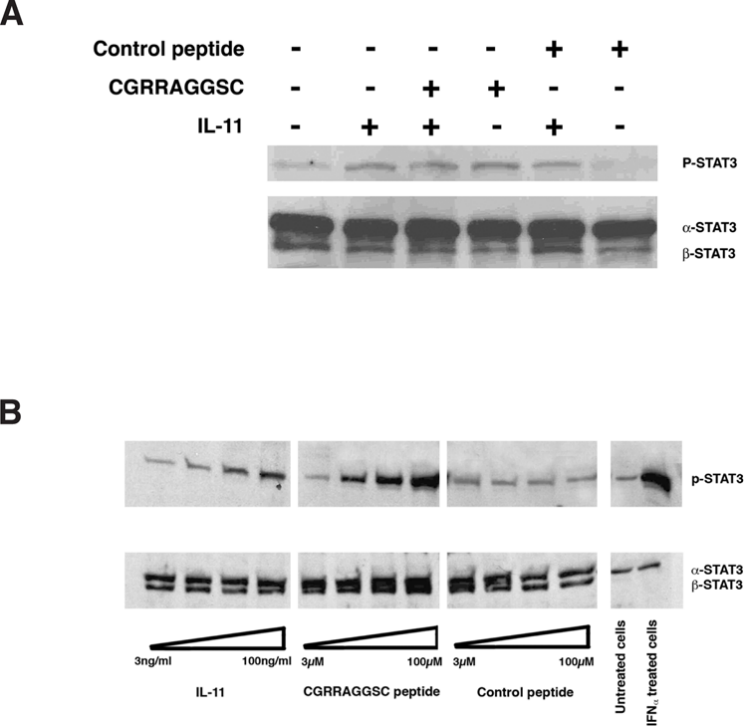
The IL-11-Like peptide CGRRAGGSC induces STAT3 activation upon binding to IL-11Rα in a concentration-dependent manner. (A) Proliferation of human TF-1 leukemia cells induced by CGRRAGGSC is associated with STAT3 phosphorylation, as assessed with an anti-STAT3-P-Tyr^705^ antibody. (B) CGRRAGGSC peptide-induced activation of STAT3 is concentration dependent. No effect is observed with a control peptide. To avoid inter-experimental variation, the lysate was divided in two: one-half of the lysate was immunoblotted with an anti-STAT3-P-Tyr^705^ antibody whereas the other half served to determine the total amount of STAT3 (as a surrogate for protein loading) with a specific anti-STAT3 antibody. Note that the commercial HeLa cell extracts serving as controls display only the ∝-STAT3 band.

IL-11 binding to IL-11Rα and glycoprotein 130 (gp130) mediates signal transduction through STAT3 activation; therefore, we next evaluated whether the CGRRAGGSC peptide might stimulate cell proliferation by activation of the same pathway. To that end, we examined ligand-mediated STAT3 activation in serum-starved TF-1 cells incubated in the presence of IL-11, CGRRAGGSC peptide, or an unrelated cyclic control peptide. Despite serum starvation, weak STAT3 (P-Tyr^705^) baseline activation was detected in unstimulated TF-1 cells (Figure 5). Twenty minutes incubation with either CGRRAGGSC or IL-11 (positive control) led to STAT3 phosphorylation (Figure 5A), which increased in a concentration-dependent manner (Figure 5B). Similar effects were observed for IL-11 in combination with CGRRAGGSC or an unrelated cyclic peptide (but not for the control peptide alone) (Figure 5A). These data uncover a potential molecular mechanism underlying CGRRAGGSC-induced proliferation via activation of the IL-11 receptor complex through STAT3 and indicate that this site within IL-11 is functionally active for binding and signal transduction.

## Discussion

**Figure 6:**
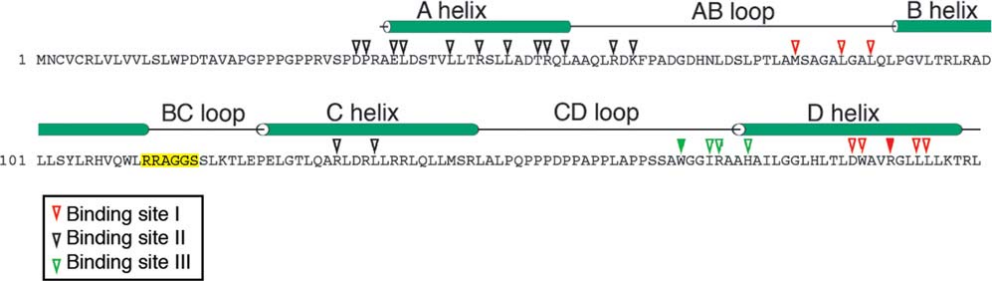
Residues identified within known human IL-11 binding sites. Arrowheads indicate human IL-11 residues predicted (through site-directed mutagenesis) to be ligand-receptor binding sites; solid colors specify residues that are critical for binding [17]–[21]. Residues 112–117 corresponding to the IL-11-like motif [1], [2] described in this work are highlighted in yellow. The basic scheme of human IL-11, as shown here, has been modified from reference [47].

Through direct selection of peptide libraries in patients [1], [37], [38], we isolated a ligand peptide mimicking IL-11 from the prostate vasculature [1] and proposed the IL-11Rα as a target during the progression of prostate cancer [2]. Here we elected to explore the structural and functional attributes of this putative protein-protein interaction because the currently known binding sites within human IL-11 [22]–[24] do not encompass the selected peptide sequence within the native protein (Figure 6).

We used complementary strategies such as site-directed mutagenesis and NMR-based studies to establish that all six residues of the RRAGGS motif are likely required for binding to IL-11Rα. Because NMR analysis did not unambiguously reveal which of the arginine residues (Arg^3^ or Arg^4^) participates in receptor binding, studies based on targeted phage alanine scanning and IL-11 site-directed mutagenesis were indicated. In both cases, Arg^4^ turned out to be the key residue. All three methods also suggested that Gly and Ser residues contribute to the level of IL-11Rα binding by the ligands. Indeed, the Ser^8^ mutation abolished the binding of CGRRAGGSC to IL-11Rα; the Δδ observed in Ser^8^ induced by binding to IL-11Rα corroborated this result.

We have attempted to generate and interpret molecular models of IL-11 based on high-resolution structural studies available for other gp130-type cytokines, including IL-6 [21], [39] and CNTF [21], [40] and on mutagenesis of IL-6, CNTF and LIF [41]–[43]. In the case of the binding site for IL-11Rα or site I, directed mutagenesis of specific residues at the C-terminus of helix D and the AB loop established that these regions participate in receptor binding and cytokine-mediated bioactivity [17]–[21], analogous to IL-6 [39]. Both locations are positioned close to each other in these models and occupy the C-terminus of the four-helix bundle structure, which is situated opposite the BC loop. Similar structural and mutagenesis-based studies on gp130-type cytokines (vIL-6, CNTF and LIF) implicated residues within the BC loop in receptor recognition and bioactivity [44]–[46]. However, such residues were part of site II or site III (for interaction with gp130 or gp130/LIF receptor), and not site I. Notably, the crystal structure of the IL-6 receptor complex hexamer demonstrated no role in IL-6 receptor binding for the BC loop, which is facing the N-terminal region of D2 in the second gp130 molecule [47]. Our data show that CGRRAGGSC is a peptide mimic of IL-11, and as such is capable of recognizing and binding to IL-11Rα to activate cell signaling and proliferation. Although it is generally assumed that the model for the cell membrane-bound IL-11 receptor complex would be similar to that for IL-6 [26], [47], certain structural characteristics in the complex subunits may actually diverge significantly. For instance, we noticed the existence of four unreported leucine-zippers within IL-11 (three of them extending from the BC loop through the helix C, Figure 6). They have no clear parallel among most other gp130-type cytokines (only OSM and CT-1 appear to contain one of such regions). Moreover, there are two IL-11Rα membrane-bound isoforms that differ by the presence or absence of a cytoplasmic domain, whereas IL-6Rα has only one form with a longer cytoplasmic tail [48]. Such isoforms could participate in the formation of a receptor complex unlike those observed in the IL-6Rα complex formation. Future detailed X-ray crystallography of the IL-11 receptor complex will further elucidate these fine structural features.

Functionally, the IL-11 mimic peptide CGRRAGGSC showed biological effects mediated by IL-11Rα (concentration-dependent stimulation of cell proliferation) both in the presence and in the absence IL-11. Furthermore, CGRRAGGSC-mediated cell proliferation in the presence of sIL-11Rα was reduced, and to a greater degree in the presence of native IL-11, but was not affected by an unrelated control receptor. Based on these findings, one might speculate on an interaction between the peptide CGRRAGGSC and the complex IL-11/IL-11Rα. Indeed, the soluble peptide CGRRAGGSC was found to induce STAT3 phosphorylation in the same manner as the IL-11 native cytokine. These results have biological precedent in other systems. Wrighton et al. [49] isolated peptides that bind to and activate the erythropoietin receptor (EPOR); crystallographic analysis of the receptor complex revealed a peptide that generated a functional symmetric arrangement of the EPOR [50]. Cwirla et al. [51] selected peptide agonists for the thrombopoietin receptor and proposed an activation mechanism. Recent studies have also shown that orientation and residence time of ligand-receptors must be considered for activation [52], [53]. Moreover, peptides selected for binding to certain receptors bind to several sites on the receptor and activation might occur through conformational changes rather than multimerization [54].

From a supra-molecular viewpoint, IL-11 receptor complex formation is in all probability intricate: the ligand cytokine (IL-11) may need to bind first to a presenting receptor subunit (IL-11Rα) and subsequently recruit a dimer of signaling subunits (i.e., gp130). Studies of the related IL-6 receptor complex support the contention that preformed inactive dimers of receptor subunits exist in cell membranes [28], [55], [56]. Previous reports determined that gp130-dimerization is not sufficient for receptor activation and that active conformational adjustment is required for a biological response [14], [57]–[59]. Therefore, it is possible that the soluble peptide CGRRAGGSC in complex with IL-11Rα might strengthen gp130-dimerization and/or induce a signaling-competent conformation.

The strength of combining various functional assays (site-directed mutagenesis of native proteins in tandem with ELISA plus peptide-alanine scanning in tandem with targeted phage binding assays) with structural studies (NMR-based spectroscopy of the peptide-receptor interaction) may overcome some of the limitations of mutational studies in the determination of binding sites of ligand-receptor interactions (such as difficult gene/protein expression, and mutant protein misfolding or steric hindrance).

In summary, we have shown that (i) the RRAGGS sequence (corresponding to a site within human IL-11, residues 112–117) mimics a candidate protein-binding site within IL-11, (ii) the molecular interaction between the peptide CGRRAGGSC and IL-11Rα is functional, and (iii) the IL-11-like motif induces proliferation of responsive cells through IL-11Rα-mediated STAT3 activation.

## Materials and Methods

### Reagents

The following antibodies were obtained commercially: monoclonal anti-IL-11 (R&D Systems, Minneapolis, MN), polyclonal anti-IL-11Rα (Santa Cruz Biotechnology, Santa Cruz, CA), anti-glutathione S transferase (GST) (Amersham Pharmacia Biotech. Piscataway, NJ), anti-human Fc HRP-conjugated (Sigma-Aldrich, St. Louis, MO), and anti-STAT3 or anti-phospho-STAT3 (P-Tyr^705^) (Cell Signaling Technology, Beverly, MA). Recombinant proteins (rhIL-11, IL-11Rα, gp130 and LeptinR) were purchased from R&D Systems. Soluble CGRRAGGSC peptide was synthesized and cyclized at AnaSpec (San Jose, CA). Phage displaying the peptide CGRRAGGSC or its scrambled version CRGSGAGRC have been described [1], [2].

### Phage binding assay

CGRRAGGSC phage recovered in vivo served for in vitro binding to IL-11Rα and gp130 [1], [2]. IL-11Rα, gp130 or control leptin receptor (the extracellular domain fused to the Fc region of human IgG; R&D Systems) were coated on microtiter wells. [60]. Proteins were immobilized (2 µg/ml) on microtiter wells overnight at 4°C, washed twice with phosphate buffered saline (PBS), blocked PBS containing with 3% bovine serum albumin (BSA) for 2 h at room temperature, and incubated with 10^9^ TU of targeted or negative control phage (insertless). After 1 h at room temperature, wells were washed twelve times with PBS, and bound phage were recovered by bacterial infection and plated as described [1].

### NMR spectroscopy

NMR experiments were acquired on a 600 MHz spectrometer (Bruker Avance DRX). CGRRAGGSC (400 µM) chemical shift assignment was performed in 50 mM sodium phosphate buffer containing 5 mM sodium azide and 10% D_2_O. Two-dimensional spectra were collected with 1,024 transients. NOESY spectra [61] were acquired with 150 ms mixing time. Spectra were collected (4,096 F1 points, 512 F2 points). TOCSY spectra (spin-lock time = 80 ms) were acquired with MLEV-17 [62] (4,096 F1 points, 512 F2 points). Water suppression was achieved with WATERGATE [63]. One-dimensional ^1^H-NMR spectra of peptide in the presence of IL-11Rα (6 µM) were obtained. Chemical shift perturbations were followed by TOCSY and NOESY by comparison of the spectra of CGRRAGGSC (400 µM) free in solution or in the presence of IL-11Rα. Analysis of the 1D-^1^H-NMR spectra with increasing concentrations of CGRRAGGSC (up to 400 µM) revealed no significant resonance shifts (Figure S1B), data indicating that the peptide was monomeric. Sodium 2,2-dimethyl-2-silapentane-5-sufonate (70 µM) served as a standard for precise spectral overlapping.

### Protein expression and purification

IL-11 alanine-scanning mutants were expressed in *E. coli* BL21 and purified from inclusion bodies. In brief, cells were cultured in 2×YT media supplemented with 200 µg/ml carbenicillin until OD_595 nm_∼0.6, at which point GST expression was induced with 1 mM IPTG. After 4 h of induction, bacteria were collected by centrifugation (5,000 g for 10 min) and lysed with BugBuster reagent (EMD Biosciences, CA) according to the manufacturer's recommendations. GST-IL-11 fusion proteins within the inclusion body fractions were collected by centrifugation at 28,000 g for 30 min and washed with 8 M urea in PBS. The purified inclusion bodies were solubilized in 6 M guanidine-HCl, 50 mM Tris-HCl pH 8, 100 mM NaCl, 10 mM EDTA, and 10 mM DTT at 0.5 to 1 mg/ml (solubilization buffer). Purified recombinant proteins were analyzed by staining with Coomassie Brilliant Blue, and Western blot with anti-IL-11 and anti-GST antibodies. To confirm the proper folding of the IL-11-GST mutants, Western blot with one polyclonal anti-IL-11 and two monoclonal anti-IL-11 antibodies were performed (data not shown).

### IL-11 site-directed mutagenesis

The human IL-11 cDNA clone was obtained from Invitrogen (Clone ID 4476678). We designed specific PCR primers containing *BamH*I and *Not*I for PCR amplification and directional cloning of the IL-11 open reading frame in the pGEX4T-1 prokaryotic expression vector (Amersham). The GST-IL-11 clone was sequenced to confirm the fusion of both proteins. We used the GST-IL-11 plasmid as a template for the generation of the IL-11 mutants. PCR mutagenesis was performed in a reaction volume of 50 µl that included 1 µL plasmid DNA (20 ng/µl), 10 pmol primers, 200 µM dNTPs, and 2.5 U *Pfu* Ultra high fidelity DNA polymerase (Stratagene, La Jolla, CA). Sense and antisense primers were designed to substitute nucleotides and to introduce an alanine residue. The oligonucleotides used for IL-11 mutagenesis were: R112A, 5′-CTGCGGCACGTGCAGTGGCTGGCCCGGGCAGGTGGCTCTTCCCTG-3′; R113A, 5′-CGGCACGTGCAGTGGCTGCGCGCGGCAGGTGGCCTCTCCCTGAAG-3′; G115A, 5′-GTGCAGTGGCTGCGCCGGGCAGCTGGCTCTTCCCTGAAGACCCTG-3′; G116A, 5′-CAGTGGCTGCGCCGGGCAGGTGCCTCTTCCCTGAAGACCCTGGAG-3′; S117A, 5′-TGGCTGCGCCGGGCAGGTGGCGCTTCCCTGAAGACCCTGGAGCCC-3′. A preliminary denaturation step at 95°C for 3 min was followed by 20 cycles of PCR. These PCR cycles consisted of 30 s of denaturation at 95°C, 1 min of annealing at 65°C and 10 min of extension at 72°C. Finally the PCR product was treated with 1 µl DpnI endonuclease for 2 h at 37°C, and 2 µl DpnI-digested DNA was transformed into 50 µl DH5α competent bacteria. Colonies were picked for plasmid isolation (QIAGEN, Valencia, CA), and nucleotide sequence analysis was performed at the DNA Core Facility, M.D. Anderson Cancer Center (Houston, TX).

### ELISA assay

GST fusion proteins of wild-type IL-11, alanine scan mutants of amino acids 112 to 117 of IL-11, or GST alone were coated overnight onto 96-well plates at 5 µg/ml. Recombinant IL-11 was added at 2 µg/ml. Wells were subsequently rinsed with wash buffer (0.1% Tween in PBS), followed by incubation with a blocking buffer (3% BSA in PBS) for 1 h at room temperature (RT). IL-11Rα (1 µg/ml) was added and incubated for 2 h. Binding was detected with anti-human Fc HRP-conjugated antibody (1∶5000). To confirm adequacy of coating, we performed parallel ELISAs with anti-GST antibody (1∶1500, data not shown). Leptin receptor was used as Fc protein and binding specificity control.

### Alanine-scanning mutagenesis of phage-displayed peptides

DNA sequences encoding residue substitutions were introduced into the peptide CGRRAGGSC as indicated. Self-annealing synthetic oligonucleotides (Sigma-Genosys, Woodlands, TX) were combined and resuspended in annealing buffer (10 mM Tris-HCl pH 8.0, containing 100 mM NaCl and 1 mM EDTA) at 10 nM. Oligonucleotide mixtures were heated to 100°C for 5 min and cooled down to RT over a 30 min. Self-annealed oligonucleotides were directly cloned into *Sfi*I-digested fUSE5 vector by using T4 DNA ligase (Roche Applied Science, Indianapolis, IN). Alternatively, 500 ng of the synthetic oligonucleotide templates encoding the displayed peptides were converted to double-stranded DNA by PCR amplification with the primer set 5′-GTGAGCCGGCTGCCC-3′ and 5′-TTCGGCCCCAGCGGC-3′, and Taq-DNA polymerase (Promega, Madison, WI). Double-stranded PCR products were treated with the restriction enzyme *Bgl*II and were cloned into the *Sfi*I-digested fUSE5 vector. In all cases, plasmids were transformed into competent DH5α *E. coli*, and DNA was verified by sequencing. Oligonucleotides used for the mutational studies were: CARRAGGSC-forward, 5′-GGGCTTGTGCGCGGAGGGCGGGCGGTTCGTGTGGGGCCGCTG-3′; CARRAGGSC-reverse, 5′-CGGCCCCACACGAACCGCCCGCCCTCCGCGCACAAGCCCCGT-3′; CGARAGGSC-forward, 5′-GGGCTTGTGGGGCGAGGGCGGGCGGTTCGTGTGGGGCCGCTG-3′; CGARAGGSC-reverse, 5′-CGGCCCCACACGAACCGCCCGCCCTCGCCCCACAAGCCCCGT-3′; CGRAAGGSC-forward, 5′-GGGCTTGTGGGCGGGCGGCGGGCGGTTCGTGTGGGGCCGCTG-3′; CGRAAGGSC-reverse, 5′-CGGCCCCACACGAACCGCCCGCCGCCCGCCCACAAGCCCCGT-3′; CGRRAAGSC-forward, 5′-GGGCTTGTGGGCGGAGGGCGGCGGGTTCGTGTGGGGCCGCTG-3′; CGRRAAGSC-reverse, 5′-CGGCCCCACACGAACCCGCCGCCCTCCGCCCACAAGCCCCGT-3′; CGRRAGASC-forward, 5′-GGGCTTGTGGGCGGAGGGCGGGCGCGTCGTGTGGGGCCGCTG-3′; CGRRAGASC-reverse, 5′-CGGCCCCACACGACGCGCCCGCCCTCCGCCCACAAGCCCCGT-3′; CGRRAGGAC-forward, 5′-GGGCTTGTGGGCGGAGGGCGGGCGGTGCGTGTGGGGCCGCTG-3′; CGRRAGGAC-reverse, 5′-CGGCCCCACACGCACCGCCCGCCCTCCGCCCACAAGCCCCGT-3′.

Phage binding with the alanine-scanning displaying phage were performed as described above. Insertless and IL-11-like phage served as a negative and positive control. All experiments were performed in duplicate and repeated at least four times with similar results.

### Cell culture and proliferation

Growth factor-dependent erythroleukemia cells (TF-1) were obtained from the American Type Culture Collection (ATCC; Manassas, VA) and were cultured in RPMI-1640 containing L-glutamine, sodium bicarbonate, glucose, HEPES, sodium pyruvate, 10% FBS, and 2 ng/ml recombinant GM-CSF (R&D Systems). EF43.*fgf-4*, which served as control cells unless otherwise specified, were maintained in DMEM containing high glucose and 10% FBS. Cell proliferation was measured by metabolic conversion of WST-1 to formazan (Roche). Cells were incubated in 96-well dishes (30,000 cells/well) with RPMI-1640 containing 1% FBS plus 3, 10, 30, 100, 300, or 1,000 µM CGRRAGGSC and/or recombinant human IL-11 (0.5 ng/ml) at 37°C for 72 h. GM-CSF (0.01 ng/ml) served as a positive control for proliferation, and an unrelated peptide (CGSPGWVRC) was used as a negative control. Inhibition of CGRRAGGSC-induced cell proliferation was assessed in the presence of 100 ng/ml of either human sIL-11Rα or soluble leptin receptor (negative control).

### STAT3 activation assay

TF-1 cells (750,000 cells/well, 24-well dishes) were incubated 18–24 hr in serum-free RPMI-1640. Subsequently, CGRRAGGSC or negative control peptide (100 µM each), either alone or in combination with human IL-11 (10 ng/ml), was added for 15 min. For experiments measuring concentration-dependence, the concentrations were 3, 10, 30 and 100 µM (peptides) or 3, 10, 30 and 100 ng/ml (IL-11). Cells were centrifuged, extracts were solubilized with Laemmli sample buffer containg ß-mercatptoethanol, lysates were resolved on 4–20% gradient SDS-PAGE gels and bands were transferred onto nitrocellulose membranes (Bio-Rad, Hercules, CA). Membranes were blocked and subsequently incubated with anti-STAT3 or anti-phospho-STAT3 (P-Tyr^705^) antibodies (1∶1,000) followed by HRP-coupled anti-rabbit antibody (1∶2000; Bio-Rad). Detection was by ECL (Amersham). Commercial HeLa cell extracts treated or not treated with interferon-α (Cell Signaling) served as controls.

## Supporting Information

Figure S1 The IL-11 mimic peptide CGRRAGGSC is multiconformational and monomeric (A) Amide region of the 1D-1H-NMR of CGRRAGGSC peptide (400 µM) at 25°C and at 5°C. (B) The amide regions of 1D-1H-NMR spectra under increasing concentrations of the IL-11-like peptide CGRRAGGSC at 25°C are shown. The presence of broad lines indicates peptide conformational exchange. No peptide oligomerization induced by increasing concentrations of CGRRAGGSC (up to 400 µM) was observed under the experimental conditions used.
(0.53 MB TIF)

Table S1 Chemical shift for the IL-11-like peptide
(0.05 MB DOC)

Table S2 Chemical shift for new resonances in CGRRAGGSC
(0.03 MB DOC)

## References

[1] Arap W, Kolonin MG, Trepel M, Lahdenranta J, Cardó-Vila M (2002). Steps toward mapping the human vasculature by phage display.. Nat Med.

[2] Zurita AJ, Troncoso P, Cardó-Vila M, Logothetis CJ, Pasqualini R (2004). Combinatorial screenings in patients: the interleukin-11 receptor alpha as a candidate target in the progression of human prostate cancer.. Cancer Res.

[3] Arap W, Pasqualini R, Ruoslahti E (1998). Cancer treatment by targeted drug delivery to tumor vasculature in a mouse model.. Science.

[4] Ellerby HM, Arap W, Ellerby LM, Kain R, Andrusiak R (1999). Anti-cancer activity of targeted pro-apoptotic peptides.. Nat Med.

[5] Koivunen E, Arap W, Valtanen H, Rainisalo A, Medina OP (1999). Tumor targeting with a selective gelatinase inhibitor.. Nat Biotechnol.

[6] Giordano RJ, Cardó-Vila M, Lahdenranta J, Pasqualini R, Arap W (2001). Biopanning and rapid analysis of selective interactive ligands.. Nat Med.

[7] Marchiò S, Lahdenranta J, Schlingemann RO, Valdembri D, Wesseling P (2004). Aminopeptidase A is a functional target in angiogenic blood vessels.. Cancer Cell.

[8] Kolonin MG, Saha PK, Chan L, Pasqualini R, Arap W (2004). Reversal of obesity by targeted ablation of adipose tissue.. Nat Med.

[9] Arap MA, Lahdenranta J, Mintz PJ, Hajitou A, Sarkis AS (2004). Cell surface expression of the stress response chaperone GRP78 enables tumor targeting by circulating ligands.. Cancer Cell.

[10] Hajitou A, Trepel M, Lilley CE, Soghomonyan S, Alauddin MM (2006). A hybrid vector for ligand-directed tumor targeting and molecular imaging.. Cell.

[11] Kang Y, Siegel PM, Shu W, Drobnjak M, Kakonen SM (2003). A multigenic program mediating breast cancer metastasis to bone.. Cancer Cell.

[12] Campbell CL, Jiang Z, Savarese DM, Savarese TM (2001). Increased expression of the interleukin-11 receptor and evidence of STAT3 activation in prostate carcinoma.. Am J Pathol.

[13] Du X, Williams DA (1997). Interleukin-11: review of molecular, cell biology, and clinical use.. Blood.

[14] http://www.fda.gov/cder/biologics/products/opregen112597.htm. Center for Drug Evaluation and Research. U.S. Food and Drug Administration, Drug Information

[15] Bravo J, Heath JK (2000). Receptor recognition by gp130 cytokines.. Embo J.

[16] Heinrich PC, Behrmann I, Haan S, Hermanns HM, Muller-Newen G (2003). Principles of interleukin (IL)-6-type cytokine signalling and its regulation.. Biochem J.

[17] Tacken I, Dahmen H, Boisteau O, Minvielle S, Jacques Y (1999). Definition of receptor binding sites on human interleukin-11 by molecular modeling-guided mutagenesis.. Eur J Biochem.

[18] Czupryn MJ, McCoy JM, Scoble HA (1995). Structure-function relationships in human interleukin-11. Identification of regions involved in activity by chemical modification and site-directed mutagenesis.. J Biol Chem.

[19] Czupryn M, Bennett F, Dube J, Grant K, Scoble H (1995). Alanine-scanning mutagenesis of human interleukin-11: identification of regions important for biological activity.. Ann N Y Acad Sci.

[20] Harmegnies D, Wang XM, Vandenbussche P, Leon A, Vusio P (2003). Characterization of a potent human interleukin-11 agonist.. Biochem J.

[21] Barton VA, Hudson KR, Heath JK (1999). Identification of three distinct receptor binding sites of murine interleukin-11.. J Biol Chem.

[22] Dahmen H, Horsten U, Kuster A, Jacques Y, Minvielle S (1998). Activation of the signal transducer gp130 by interleukin-11 and interleukin-6 is mediated by similar molecular interactions.. Biochem J.

[23] Kurth I, Horsten U, Pflanz S, Dahmen H, Kuster A (1999). Activation of the signal transducer glycoprotein 130 by both IL-6 and IL-11 requires two distinct binding epitopes.. J Immunol.

[24] Schleinkofer K, Dingley A, Tacken I, Federwisch M, Muller-Newen G (2001). Identification of the domain in the human interleukin-11 receptor that mediates ligand binding.. J Mol Biol.

[25] Boulanger MJ, Chow DC, Brevnova EE, Garcia KC (2003). Hexameric structure and assembly of the interleukin-6/IL-6 alpha-receptor/gp130 complex.. Science.

[26] Muller-Newen G (2003). The cytokine receptor gp130: faithfully promiscuous.. Sci STKE PE40.

[27] Hermanns HM, Muller-Newen G, Heinrich PC, Haan S (2005). Bow to your partner for signaling.. Nat Struct Mol Biol.

[28] Schroers A, Hecht O, Kallen KJ, Pachta M, Rose-John S (2005). Dynamics of the gp130 cytokine complex: a model for assembly on the cellular membrane.. Protein Sci.

[29] Grotzinger J, Kernebeck T, Kallen KJ, Rose-John S (1999). IL-6 type cytokine receptor complexes: hexamer, tetramer or both?. Biol Chem.

[30] Giordano RJ, Anobom CD, Cardó-Vila M, Kalil J, Valente AP (2005). Structural basis for the interaction of a vascular endothelial growth factor mimic peptide motif and its corresponding receptors.. Chem Biol.

[31] Valente AP, Miyamoto CA, Almeida FC (2006). Implications of protein conformational diversity for binding and development of new biological active compounds.. Curr Med Chem.

[32] Pires JR, Taha-Nejad F, Toepert F, Ast T, Hoffmuller U (2001). Solution structures of the YAP65 WW domain and the variant L30 K in complex with the peptides GTPPPPYTVG, N-(n-octyl)-GPPPY and PLPPY and the application of peptide libraries reveal a minimal binding epitope.. J Mol Biol.

[33] Cruzeiro-Silva C, Gomes-Neto F, Tinoco LW, Cilli EM, Barros PV (2007). Structural biology of membrane-acting peptides: conformational plasticity of anticoccidial peptide PW2 probed by solution NMR.. Biochim Biophys Acta.

[34] Henzler-Wildman K, Kern D (2007). Dynamic personalities of proteins.. Nature.

[35] Henzler-Wildman KA, Thai V, Lei M, Ott M, Wolf-Watz M (2007). Intrinsic motions along an enzymatic reaction trajectory.. Nature.

[36] Fourcin M, Chevalier S, Lebrun JJ, Kelly P, Pouplard A (1994). Involvement of gp130/interleukin-6 receptor transducing component in interleukin-11 receptor.. Eur J Immunol.

[37] Pentz RD, Flamm AL, Pasqualini R, Logothetis CJ, Arap W (2003). Revisiting ethical guidelines for research with terminal wean and brain-dead participants.. Hastings Cent Rep.

[38] Pentz RD, Cohen CB, Wicclair M, DeVita MA, Flamm AL (2005). Ethics guidelines for research with the recently dead.. Nat Med.

[39] Somers W, Stahl M, Seehra JS (1997). 1.9 A crystal structure of interleukin 6: implications for a novel mode of receptor dimerization and signaling.. Embo J.

[40] McDonald NQ, Panayotatos N, Hendrickson WA (1995). Crystal structure of dimeric human ciliary neurotrophic factor determined by MAD phasing.. Embo J.

[41] Panayotatos N, Radziejewska E, Acheson A, Somogyi R, Thadani A (1995). Localization of functional receptor epitopes on the structure of ciliary neurotrophic factor indicates a conserved, function-related epitope topography among helical cytokines.. J Biol Chem.

[42] Simpson RJ, Hammacher A, Smith DK, Matthews JM, Ward LD (1997). Interleukin-6: structure-function relationships.. Protein Sci.

[43] Hudson KR, Vernallis AB, Heath JK (1996). Characterization of the receptor binding sites of human leukemia inhibitory factor and creation of antagonists.. J Biol Chem.

[44] Ciapponi L, Graziani R, Paonessa G, Lahm A, Ciliberto G (1995). Definition of a composite binding site for gp130 in human interleukin-6.. J Biol Chem.

[45] Chow D, He X, Snow AL, Rose-John S, Garcia KC (2001). Structure of an extracellular gp130 cytokine receptor signaling complex.. Science.

[46] Kallen KJ, Grotzinger J, Lelievre E, Vollmer P, Aasland D (1999). Receptor recognition sites of cytokines are organized as exchangeable modules. Transfer of the leukemia inhibitory factor receptor-binding site from ciliary neurotrophic factor to interleukin-6.. J Biol Chem.

[47] Boulanger MJ, Cho DC, Brevnova E, Martick M, Sandford G (2004). Molecular mechanisms for viral mimicry of a human cytokine: activation of gp130 by HHV-8 interleukin-6.. J Mol Biol.

[48] Lebeau B, Montero Julian FA, Wijdenes J, Muller-Newen G, Dahmen H (1997). Reconstitution of two isoforms of the human interleukin-11 receptor and comparison of their functional properties.. FEBS Lett.

[49] Wrighton NC, Farrell FX, Chang R, Kashyap AK, Barbone FP (1996). Small peptides as potent mimetics of the protein hormone erythropoietin.. Science.

[50] Livnah O, Stura EA, Johnson DL, Middleton SA, Mulcahy LS (1996). Functional mimicry of a protein hormone by a peptide agonist: the EPO receptor complex at 2.8 A.. Science.

[51] Cwirla SE, Balasubramanian P, Duffin DJ, Wagstrom CR, Gates CM (1997). Peptide agonist of the thrombopoietin receptor as potent as the natural cytokine.. Science.

[52] Livnah O, Johnson DL, Stura EA, Farrell FX, Barbone FP (1998). An antagonist peptide-EPO receptor complex suggests that receptor dimerization is not sufficient for activation.. Nat Struct Biol.

[53] Seubert N, Royer Y, Staerk J, Kubatzky KF, Moucadel V (2003). Active and inactive orientations of the transmembrane and cytosolic domains of the erythropoietin receptor dimer.. Mol Cell.

[54] Pillutla RC, Hsiao KC, Beasley JR, Brandt J, Ostergaard S (2002). Peptides identify the critical hotspots involved in the biological activation of the insulin receptor.. J Biol Chem.

[55] Varghese JN, Moritz RL, Lou MZ, Van Donkelaar A, Ji H (2002). Structure of the extracellular domains of the human interleukin-6 receptor alpha -chain.. Proc Natl Acad Sci U S A.

[56] Schuster B, Meinert W, Rose-John S, Kallen KJ (2003). The human interleukin-6 (IL-6) receptor exists as a preformed dimer in the plasma membrane.. FEBS Lett.

[57] Greiser JS, Stross C, Heinrich PC, Behrmann I, Hermanns HM (2002). Orientational constraints of the gp130 intracellular juxtamembrane domain for signaling.. J Biol Chem.

[58] Muller-Newen G, Kuster A, Wijdenes J, Schaper F, Heinrich PC (2000). Studies on the interleukin-6-type cytokine signal transducer gp130 reveal a novel mechanism of receptor activation by monoclonal antibodies.. J Biol Chem.

[59] Skiniotis G, Boulanger MJ, Garcia KC, Walz T (2005). Signaling conformations of the tall cytokine receptor gp130 when in complex with IL-6 and IL-6 receptor.. Nat Struct Mol Biol.

[60] Smith GP, Scott JK (1993). Libraries of peptides and proteins displayed on filamentous phage.. Meth Enzymol.

[61] Piotto M, Saudek V, Sklenar V (1992). Gradient-tailored excitation for single-quantum NMR spectroscopy of aqueous solutions.. J Biomol NMR.

[62] Bax A, Davis DG (1985). Mlev-17-Based Two-Dimensional Homonuclear Magnetization Transfer Spectroscopy.. J Magn Reson.

[63] Sklenar V, Piotto M, Leppik R, Saudek V (1993). Gradient-tailored water suppression for H-1-N-15 Hsqc experiments optimized to retain full sensitivity.. J Magn Reson Series A.

