# Supplementary figures and images for: A Ligand Peptide Motif Selected from a Cancer Patient Is a Receptor-Interacting Site within Human Interleukin-11
Source: PLoS One. 2008 Oct 20;3(10):e3452. doi: 10.1371/journal.pone.0003452 (PMC2565473; doi:10.1371/journal.pone.0003452)

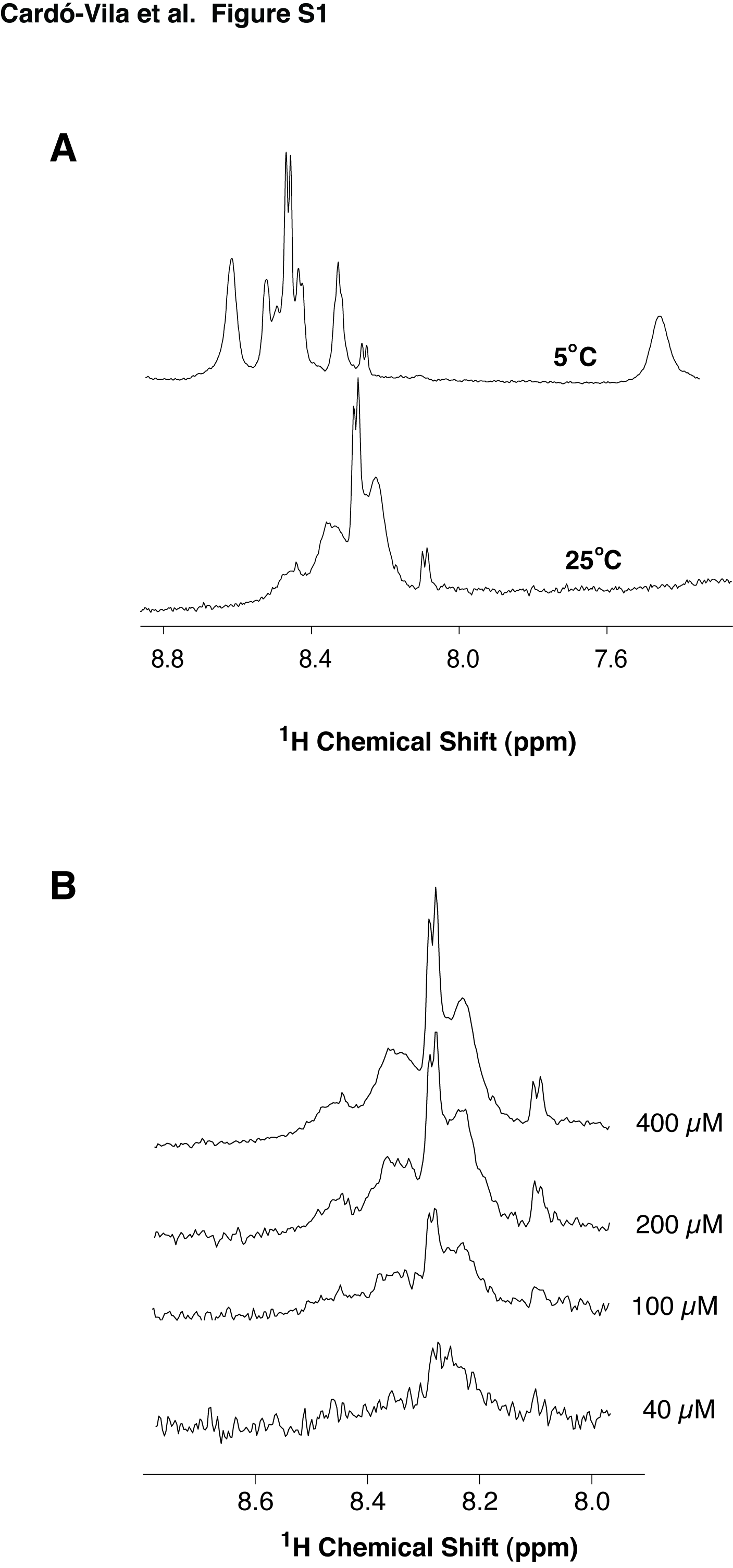

Supplement: Figure S1 — The IL-11 mimic peptide CGRRAGGSC is multiconformational and monomeric (A) Amide region of the 1D-1H-NMR of CGRRAGGSC peptide (400 µM) at 25°C and at 5°C. (B) The amide regions of 1D-1H-NMR spectra under increasing concentrations of the IL-11-like peptide CGRRAGGSC at 25°C are shown. The presence of broad lines indicates peptide conformational exchange. No peptide oligomerization induced by increasing concentrations of CGRRAGGSC (up to 400 µM) was observed under the experimental conditions used. (0.53 MB TIF) [file pone.0003452.s001.tif]
