# Supplementary material for: A Ligand Peptide Motif Selected from a Cancer Patient Is a Receptor-Interacting Site within Human Interleukin-11
Source: PLoS One. 2008 Oct 20;3(10):e3452. doi: 10.1371/journal.pone.0003452 (PMC2565473; doi:10.1371/journal.pone.0003452)
Supplement: Table S1 — Chemical shift for the IL-11-like peptide (0.05 MB DOC) [file pone.0003452.s002.doc]

**Table S1. Chemical shift for the IL-11-like peptide**

| **Residue** | **HN** | **Hα** | **Hβ** | **Others** |
| --- | --- | --- | --- | --- |
|  |  |  |  |  |
| **Cys^1^** | 8.080 | 4.538 | 3.270; 3.072 |  |
| **Cys^1/9^** | - | 3.940 | 3.106 |  |
|  |  |  |  |  |
| **Gly^2^** | - | 3.924 | - |  |
|  |  |  |  |  |
| **Arg^3^** | - | 4.329 | 1.868; 1.774 | γCH_3_: 1.621; CH_2_ : 3.190 |
|  |  |  |  |  |
| **Arg^4^** | - | 4.278 | 1.868; 1.774 | γCH_3_: 1.621; CH_2_: 3.190 |
|  |  |  |  |  |
| **Ala^5^** | - | 4.304 | 1.392 |  |
| **Ala^5^** | - | 4.100 | 1.308 |  |
|  |  |  |  |  |
| **Gly^6^** | 8.236 | 4.101; 3.856 |  |  |
|  |  |  |  |  |
| **Gly^7^** | 8.212 | 3.945; 3.937 |  |  |
|  |  |  |  |  |
| **Ser^8^** | - | 4.545 | 3.879 |  |
|  |  |  |  |  |
| **Cys^9^** | 8.268 | 4.500 | 3.300; 3.064 |  |
|  |  |  |  |  |

CGRRAGGSC (400 μM) at 25 °C.
