# Supplementary material for: A Ligand Peptide Motif Selected from a Cancer Patient Is a Receptor-Interacting Site within Human Interleukin-11
Source: PLoS One. 2008 Oct 20;3(10):e3452. doi: 10.1371/journal.pone.0003452 (PMC2565473; doi:10.1371/journal.pone.0003452)
Supplement: Table S2 — Chemical shift for new resonances in CGRRAGGSC (0.03 MB DOC) [file pone.0003452.s003.doc]

**Table S2. Chemical shift for new resonances in CGRRAGGSC**

| **Residue** | **HN** | **Hα** | **Hβ** | **Others** |
| --- | --- | --- | --- | --- |
|  |  |  |  |  |
| **Cys^1/9^*** | 8.220 | 4.513 | 3.293; 3.066 |  |
| **Cys^1/9^*** | - | 3.991 | 3.156; 2.996 |  |
|  |  |  |  |  |
| **Arg^3/4^*** | 7.877 | 4.202 | 1.851; 1.720 | γCH_3_: 1.587; CH_2_: 3.182 |
|  |  |  |  |  |
| **Ala^5^*** | - | 4.395 | 1.418 |  |
|  |  |  |  |  |
